# Building Lay Society Knowledge and Education for Health Technology Assessment and Policy Engagement: Case of CFTR Modulator Access in Brazil

**DOI:** 10.3390/healthcare13161996

**Published:** 2025-08-14

**Authors:** Verônica Stasiak Bednarczuk de Oliveira, Marise Basso Amaral, Mariana Camargo, Miquéias Lopes-Pacheco

**Affiliations:** 1 United for Life Institute—Cystic Fibrosis, Rare and Respiratory Diseases, Curitiba 80420-130, PR, Brazil; 2 Department of Pharmaceutical Sciences, Federal University of Paraná, Curitiba 80210-170, PR, Brazil; 3 Department of Society, Education, and Knowledge, Faculty of Education, Fluminense Federal University, Niterói 24210-201, RJ, Brazil; 4 Department of Surgery, Division of Urology, São Paulo Federal University, São Paulo 04021-001, SP, Brazil; 5 Department of Pediatrics, Center for Cystic Fibrosis and Airway Disease Research, Emory University School of Medicine, Atlanta, GA 30322, USA

**Keywords:** cystic fibrosis, health policy, healthcare disparities, patient advocacy, personalized care, precision medicine, public health education, rare disease, stakeholder engagement

## Abstract

The health technology assessment (HTA) is a multidisciplinary process utilized to determine the clinical, economic, social, and ethical value of new health technologies before they are incorporated into healthcare systems. In the case of rare diseases, such as Cystic Fibrosis (CF), challenges arise due to limited evidence and high treatment costs. Indeed, although CF transmembrane conductance regulator (CFTR) modulators are breakthrough therapies for CF, their incorporation into public health systems has been complex with considerable challenges, especially in low- and middle-income countries. This article presents a descriptive and exploratory case study of the regulatory and policy journey for CFTR modulators to be approved in Brazil. Based on a narrative review and document analysis, we highlight the importance of building lay society knowledge to shape policy decisions and promote equitable access to innovative therapies. In parallel, we critically reflect on the HTA process and highlight efforts in the training, education, and coordination required to enable meaningful public engagement and landmark achievements.

## 1. Introduction

Health technologies play an essential role in modern healthcare by preventing, diagnosing, or treating medical conditions, thus improving the quality of life and reducing morbidity and mortality rates [1]. These technologies encompass a wide range of products and services, including diagnostic tools, digital health and information systems, medical devices, surgical procedures, and new medications. Nevertheless, before such technologies become available to patients, they are evaluated in a multidisciplinary process—termed the health technology assessment (HTA)—that utilizes well-defined methods to determine medical, economic, social, and ethical implications. Collectively, it aims to provide evidence-based information to ensure that new technology is safe, efficient, and cost-effective, thus assisting decision-making to promote an equitable, efficient, and high-quality health system [1,2]. However, new health technologies often bring new dilemmas, with the HTA facing significant challenges when considering rare diseases due to the specific nature of these conditions. Among >7000 known rare diseases, Cystic Fibrosis (CF) is one of the most common [3,4]. CF is a life-threatening autosomal recessive inherited disease caused by mutations in the gene encoding the CF transmembrane conductance regulator (CFTR) protein [5,6,7], a chloride and bicarbonate channel residing at the apical membrane of the secretory epithelia. The loss of the CFTR activity causes a wide range of clinical manifestations, with the progressive deterioration of the lung function being the major cause of morbidity and mortality in people with CF (PwCF).

Since the disease was first described in 1938 [8], symptomatic therapies have been the mainstay of CF management and healthcare. These include numerous daily medications to assist in airway clearance, combat inflammation and infections, and provide nutritional supplementation. Together with an early diagnosis through the implementation of national newborn screening programs, the CF life expectancy was significantly enhanced, although these individuals are still susceptible to considerable physical and psycho-emotional burdens, which have a detrimental impact on their quality of life [9,10]. The progress in unraveling the pathological mechanisms underlying the disease and the development of technologies to facilitate the drug discovery process resulted in the identification of small molecules targeting the fundamental defects in the mutant CFTR protein [11,12]. These new medications—termed CFTR modulators—can rescue CFTR folding and trafficking to the apical cellular membrane (called correctors) or enhance its activity (called potentiators) and have provided a life-changing perspective for genotype-eligible PwCF [13,14].

Among the clinically approved CFTR modulators, monotherapy with the potentiator ivacaftor (IVA) [15] and the triple combination composed by the correctors elexacaftor (ELX) and tezacaftor (TEZ) plus the IVA are considered “highly effective” modulator therapies [16,17]. More recently, a novel triple combination composed by the correctors vanzacaftor (VNZ) and TEZ plus the potentiator deutivacaftor (dIVA) demonstrated similar therapeutic efficacy [18]. In short-term clinical trials and observational and real-world studies, the treatment with these CFTR modulators was demonstrated to improve the lung function, pulmonary exacerbations, and nutritional status [15,16,17,18,19]. In longer-term studies, CFTR modulators continued to reduce CF signs and symptoms, while enhancing the quality of life with an estimated increase in life expectancy [19,20,21]. Indeed, an extensive analysis of U.K. and U.S. registries evidenced sustained clinical benefits for PwCF taking IVA monotherapy [22]. Another study estimated that in 10 years, the treatment with ELX/TEZ/IVA (ETI) may reduce the mortality rate by ~15% and increase the life expectancy by ~9.2 years [23].

While CFTR modulators are more widely available in high-income countries, access to these transformative therapies remains a challenge in low- and middle-income countries [24,25]. Some relevant factors that contribute to these disparities include the monopoly and high price of these medications, restrictive patent laws preventing the production or importation of generic alternatives (or through voluntary license), and the relatively low interest from pharmaceutical companies in engaging with regions having a smaller number of diagnosed PwCF [25,26,27,28]. For instance, IVA monotherapy was first approved by the U.S. Food and Drug Administration (FDA) in early 2012 [29], but its approval in Brazil by the Brazilian Health Regulatory Agency (ANVISA) only occurred in 2018 [30] and took two additional years for its incorporation into the Brazilian Unified Health Care System (SUS) for PwCF ≥6 years and harboring CFTR variants with gating defects [31]. Notably, IVA dispensing for genotype-eligible PwCF in Brazil only started in October 2022—i.e., 10 years after its first clinical approval [32].

Likewise, ETI was approved by the FDA in late 2019 [33], and its approval by the ANVISA only came in early 2022 [34], taking an additional year for its incorporation into the SUS for PwCF ≥ 6 years and harboring at least one copy of F508del, the most frequent CF-causing variant. ETI dispensing by the SUS started in May 2024—i.e., ~5 years after its first clinical approval [35]. Such delayed access to these transformative therapies has significant detrimental implications, as their early initiation in life may have a greater impact on long-term outcomes or even prevent damage to affected organs, which is estimated to considerably improve the life expectancy [36,37].

To explore these dynamics in depth, this article presents a descriptive case study of the regulatory and policy journey for CFTR modulators to be approved by the ANVISA and incorporated into the SUS. Based on a narrative review and documentary analysis, we aim to examine how education, capacity-building, and civil society engagement contributed to a successful HTA process and policy decision. Our study is structured in three main sections. Firstly, we provide context on the CF diagnosis and access to modulator therapies: from global cases to Brazil, outlining the regulatory and policy timeline that led to the incorporation of modulators in the country. Secondly, we examine the regulatory approval process, HTA, and health technology incorporation in Brazil, including the specific challenges of conducting HTAs for rare diseases, along with the strategies of civil society engagement and capacity-building that supported the process. Lastly, we reflect on broader implications for equitable access to innovative therapies in rare diseases, particularly in low- and middle-income countries, and address ethics in the HTA, which is a critical topic that needs further attention. By analyzing these multiple factors, this article offers insights into the collective mobilization that led to current milestones in CF care in Brazil.

## 2. Methods

This is an exploratory and descriptive case study focused on the incorporation of CFTR modulators into the Brazilian SUS and the role of civil society throughout this process. This study adopts a qualitative and interdisciplinary approach, combining narrative review and documentary analysis to critically examine the regulatory, political, and societal processes involved.

Data sources included publicly available documents and institutional reports from ANVISA and the Minister of Health, national legislation, scientific literature (searched in PubMed, Scopus, and Web of Science using terms such as “Cystic Fibrosis,” “HTA,” “CFTR modulators,” and “social participation,” in both English and Portuguese), and materials produced by patient associations. The selection covered the period from 2018 to 2024, and inclusion criteria were based on relevance to the incorporation process and publication within the study timeframe.

Data were organized and analyzed through critical documentary analysis and content interpretation, guided by HTA participation frameworks. Coding was primarily deductive, aligned with thematic categories such as stakeholder roles, types of evidence, and influence mechanisms. A triangulation of sources and methods was applied to ensure consistency and analytical rigor.

## 3. CF Diagnosis and Access to Modulator Therapies: From Global Cases to Brazil

Over 100,000 individuals are diagnosed with CF worldwide [36]. A CF diagnosis is based on signs and symptoms consistent with the disease and laboratory biomarkers confirming CFTR dysfunction. These include the assessment of immunoreactive trypsinogen in the bloodspot from newborns; the identification of CFTR pathogenic variants (called mutations) in both alleles by a genetic analysis; and abnormalities in the sweat chloride concentration (>60 mmol·L^−1^), transepithelial nasal potential difference, or intestinal current measurement [38,39]. However, it is expected that a high number of undiagnosed PwCF live in low- and middle-income countries, in which no national newborn screening program and patient registry usually exist [40]. Accordingly, recent estimates indicate that the entire CF population may be composed of over 180,000 people [24]. Of these, <30% of PwCF are taking ETI, and the majority live in high-income countries [24].

Once these drugs are demonstrated to be safe and effective in large-scale trials [15,16,17,18], the license for their clinical use is first sought by the FDA and subsequently by other national agencies, including the European Medicines Agency (EMA), the Australian Therapeutic Goods Administration (TGA), and Health Canada [41]. Table 1 depicts the currently approved CFTR modulators and their approval date by regulatory agencies in different regions. Notably, there is a gap of six and three years, respectively, from the approval of IVA and ETI by the FDA (in the U.S.) to their approval by the ANVISA (in Brazil). In several other countries (e.g., Argentina and South Africa), these drugs have not even been registered yet.

Another relevant factor in CF diagnosis and access to modulator therapies is the elevated CFTR allelic heterogeneity. Indeed, over 2100 variants have been reported in the *CFTR* gene [42], of which half are now classified as CF-causing [43]. The F508del variant occurs in >80% of CF cases in the U.S. [44], Canada [45], Australia [46], and several European countries [47], thus being a preferential target for research and drug development in CF. However, the frequency is much lower in various regions. For instance, the prevalence of this variant in Turkey is only ~23% in PwCF [48]. In the Black African population, the 2988+1G>A (legacy 3120+1G>A) is among the most common CF-causing variants and is found in ~46% of sub-Saharans with CF [49]. Around 45% of Ashkenazi Jews with CF in Israel harbor the CF-causing variant W1282X [50]. In Italy, the F508del variant is present in ~70% of CF cases, followed by N1303K and G542X, both with average frequencies of ~5% [47,51]. Such CFTR allelic variability poses substantial challenges since the identification of CFTR variants in both alleles is essential for CF diagnosis, and most countries search for a narrow number of CFTR variants in the genetic testing, which may not reflect the most prevalent ones in that specific region [52,53]. Moreover, (ultra)rare CFTR variants are usually excluded from traditional clinical trials as their designs are underpowered due to the low number of individuals, who are likely to live in different regions worldwide.

Based on in vitro data in heterologous cell models, the FDA has approved label extensions of the clinically approved modulators to >270 CFTR (ultra)rare variants [54,55], with the following studies largely confirming clinical benefits [56,57]. While this has significantly increased the number of Americans with CF who may benefit from these therapies, such an approach is not yet considered sufficient for the label extension approval by other regulatory agencies worldwide [58]. Nevertheless, a growing body of literature is demonstrating that PwCF living in underprivileged regions in the U.S. or from historically marginalized communities still exhibit worse clinical outcomes, with increasing chances of acquiring pulmonary infections or having pulmonary exacerbations, and a higher risk of mortality [59,60,61]. These outcomes have been associated with a late diagnosis due to the absence of CFTR variants in the genetic testing, poorer socioeconomic conditions, and the lack of representation of these populations in the majority of studies [62,63,64,65].

In South America, over 10,000 people are diagnosed with CF in eight out of the twelve countries [24]. However, the lack of national registries and data in the literature from all countries suggests the number of PwCF may be higher. Chile, Colombia, and Uruguay are the countries with a large, diagnosed CF population in which access to ETI is still lacking [24]. Although Argentina has no reimbursement agreement in place, it is the only country with a local pharmaceutical company that manufactures the generic version of CFTR modulator drugs [26,41]. Notably, despite a lack of competitive mechanisms, the generic ETI is available in Argentina at a price that is 85% lower compared to the original version in the U.S. [26,41].

**Figure 1:**
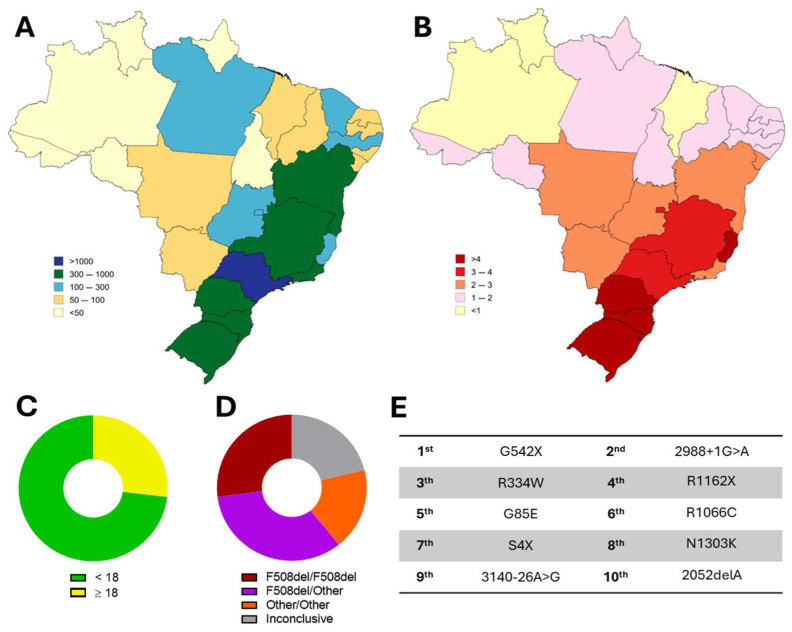
Cystic Fibrosis in Brazil. (**A**) Distribution of diagnosed PwCF according to states. (**B**) Distribution according to number of PwCF per 100,000 inhabitants. Global distribution per (**C**) age (73% pediatric and 27% adult) and (**D**) CF genotype: F508del-homozygous (27%), F508del-heterozygous (33%), carrying other (non-F508del) variants in both alleles (19%), or inconclusive (21%; i.e., only one pathogenic variant was identified or two variants with unclear consequences were identified). (**E**) Top 10 most prevalent (non-F508del) CF-causing variants. (Data from Brazilian Cystic Fibrosis Registry Report 2022).

Brazil is the fifth country with the largest CF population worldwide, with ~7000 diagnosed PwCF and an incidence of 1 in ~7500 newborns [66]. The Brazilian population is considered one of the most genetically diverse in the world, mainly due to an extensive mixture of ancestries from three distinct groups. This tri-hybrid composition is the result of over five centuries of interethnic interbreeding between South American natives, European settlers, and African slaves [67,68,69] and contributes to the differences in the incidence and prevalence of CF among Brazilian states (Figure 1) [70]. The F508del variant is found in ~60% of CF alleles in Brazil, and the remaining alleles are composed of an enormous number of CFTR variants, with several of these not listed in other global databases [43,66]. Of note, ~73% of Brazilians with CF are in the pediatric age group (i.e., ≤18 years old), and the average age of death is 22 years [66], which is much lower compared to that of high-income countries [44,45,46,47].

Until IVA monotherapy—the first CFTR modulator—was incorporated into the SUS in 2020, the national standard of care for CF was limited to symptom management therapies. At the federal level, only tobramycin, pancreatin (both available since 2016), and dornase alfa were provided [71,72]. Certain states developed specific clinical protocols and offered additional supportive treatments, such as antibiotics and vitamin supplements. These therapeutic limitations, combined with regional disparities in the access to specialized care and the genetic heterogeneity of the Brazilian CF population, contributed to significant unmet needs and suboptimal health outcomes for PwCF. Although significant challenges and barriers remain, the incorporation of CFTR modulators into standard care represents a paradigm shift, offering for the first time a disease-modifying approach to address the underlying cause of CF. Table 2 depicts features of CFTR modulators registered in the ANVISA—although not all these drugs have been incorporated into the SUS [73,74,75]. Notably, the modulator therapy VZN/TEZ/dIVA was not added to the table because it has not yet been submitted or approved by the ANVISA to date.

## 4. HTA in Brazil

### 4.1. The Regulatory Approval and Price Setting for Commercialization: The Role of the ANVISA

For a new medication to become available on the Brazilian market, it is necessary that, after the registration in its country of origin—in the case of available CFTR modulators approved by the U.S. FDA—the quality, safety, and efficacy are revised by the ANVISA, a specialized agency headquartered in the Federal District (Brasília), created by Law n. 9782/1999 to oversee the registration of any medications nationwide [76]. The ANVISA is also responsible for the sanitary control of the manufacturing and consumption of products and services subject to health surveillance, as well as the control of ports, airports, borders, and customs facilities in Brazil [77].

**Figure 2:**
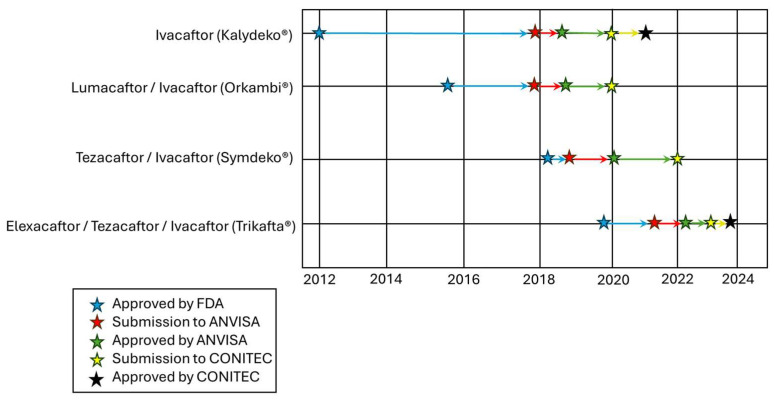
The regulatory journey timeline of clinically approved CFTR modulators in Brazil. A blue star represents the approval date by the U.S. FDA; a red star represents the date of the technology submission (by industry) to the ANVISA; a green star represents the date of approval by the ANVISA; a yellow star represents the date of the technology submission (by industry) to the CONITEC; and a black star represents the date the CONITEC approved modulators to be incorporated into the SUS. Note that LUM/IVA and TEZ/IVA were not approved by the CONITEC to be incorporated into the SUS, explaining the absence of the black star in their timeline [29,30,33,34,75,79,80].

The registration process for a new health technology initiates with the submission of a technical report containing all information about the formulation development, finished product, production, quality control of raw materials, and the product packaging, as well as clinical evidence and other related factors [78]. Additionally, the ANVISA may, at its discretion and with technical justification, require additional evidence or new studies to verify the efficacy, safety, and quality of the technology proposed for registration [78]. Figure 2 depicts the timeframe for the regulatory approval of CFTR modulator therapies in Brazil. Notably, an average gap of 41.5 months exists from the first global approval of these drugs to their approval in Brazil, although a considerable reduction in this timeframe can be observed from IVA (79 months) to ETI (27 months), respectively [29,30,33,34].

**Figure 3:**
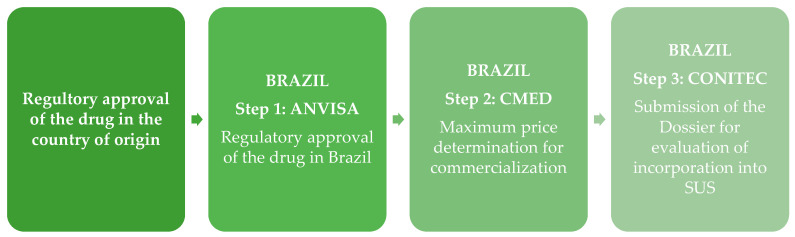
The flow of the regulatory and pricing process for medications in Brazil.

Regulatory approval by the ANVISA is a critical requirement for the commercialization of new health technologies in Brazil; however, it does not itself guarantee the subsequent incorporation of the technology into the SUS [81]. Following the approval and authorization for commercialization, the next essential step is the establishment of the maximum price of the technology, a process overseen by the Chamber for the Regulation of the Drug Market (CMED), as stipulated by Law n. 10,742 [82]. The CMED is an interministerial body, with the ANVISA serving as its executive secretariat. Its primary mandate is to regulate health technology pricing, foster market competition, monitor industry practices, and enforce penalties for non-compliance. Additionally, the CMED is responsible for setting the mandatory minimum discount for public sector purchases [83], a process that has been applied to CFTR modulators and other health technologies incorporated into the SUS. Finally, once the new technology has received health registration from the ANVISA and its price has been defined by the CMED, it can be submitted for evaluation by the National Commission for the Incorporation of Technologies into SUS (CONITEC). This collegiate body advises the Brazilian Ministry of Health on decisions regarding the incorporation, exclusion, or modification of health technologies within the SUS [84,85]. Figure 3 illustrates this process, from the regulatory approval in the country of origin to the evaluation for incorporation into the SUS, highlighting the roles of each involved entity.

In the following sub-sections, we have provided an overview of how SUS and CONITEC function and how new technology is evaluated for its incorporation into the SUS. We have also discussed how society can participate in decisions related to the incorporation of new health technologies into the SUS and how the HTA process works.

### 4.2. An Overview of the Brazilian Unified Healthcare System (SUS)

The SUS was established by the Brazilian Federal Constitution of 1988 and regulated by Law n. 8080/1990 [84], which even provides that “*health is a fundamental human right, and the State must provide the conditions necessary for its full exercise*”. It is founded on the principles of universality, comprehensiveness, and equity, thus ensuring universal and equal access to healthcare for the entire Brazilian population [84]. The SUS is considered one of the largest public health systems worldwide [86] and is structured into three main levels of care, following Ordinance 4279/2010 [87], which establishes the guidelines for the organization of the Health Care Network within the scope of the SUS, namely primary, secondary, and tertiary care. Primary care works as the gateway to the system, in which the population can access preventive health services and basic care, while specialized care is divided into secondary and tertiary care, which are medium and high complexity, respectively [87]. Secondary care involves medical specialties and more complex exams, while tertiary care corresponds to highly complex procedures, such as surgeries and specialized treatments—all completely free of charge and with no co-payment.

The management of the SUS is decentralized, with responsibilities being shared at the federal, state, and municipal levels. Although the Brazilian Ministry of Health has key responsibilities in health policies and coordinating the system, states and municipalities are fundamental to implementing these policies and managing health services in their territories [87]. Furthermore, in the context of the SUS, the participation of society in decisions related to health has been established as a constitutional guideline since its creation and has been regulated through Health Conferences and Councils [1]. The system includes mechanisms for social control in which representatives of civil society can actively participate in the planning and monitoring of SUS activities.

### 4.3. The Incorporation of a New Health Technology into the SUS

#### 4.3.1. The Role of the CONITEC

The CONITEC employs HTAs to inform decision-making processes, ensuring that the most appropriate technologies are made available to patients based on established criteria. An essential aspect of this process is the integration of patient perspectives. While there are no restrictions on who may request a new evaluation from the CONITEC, one of the primary external stakeholders seeking the evaluation of new medicines is the pharmaceutical industry [88].

When the CONITEC receives a request to evaluate a new technology, accompanied by a dossier containing relevant information about the technology, the regulatory period for the analysis begins. This period spans 180 days, with the possibility to extend for an additional 90 days. During this timeframe, in addition to reviewing the submitted documents, a preliminary meeting is held to present the technology to decision-makers. A patient also participates in a 10-minute segment, sharing personal experiences either with the technology under evaluation or the disease in question. Following this meeting, the Committee reviews the evidence, votes, and communicates its preliminary recommendation regarding the potential incorporation of the technology into the SUS, indicating whether the recommendation is favorable or unfavorable [89]. Following the initial evaluation and recommendation, the preliminary decision is subjected to a public consultation for 20 days, unless its urgency justifies a reduction to 10 days [90].

In addition to addressing the clinical, economic, and organizational aspects of the technology, the HTA should also consider the perspectives and experiences of patients and other stakeholders, thereby reconciling technical decisions with societal engagement [91]. Global HTA agencies explore various approaches to incorporating the patient perspective into their social engagement strategies, aiming to ensure that HTA processes remain patient-centered [91,92]. The public consultation serves as the mechanism through which the CONITEC facilitates the involvement of health system users, allowing them to share their real-life experiences with the technology and their diseases. Additionally, patient associations, family members, and medical societies are encouraged to submit reports contributing to informed decision-making. The applicant is also required to present a revised price proposal during the public consultation, further enhancing the inclusivity and transparency of the process.

Accordingly, the consultation period aims to enhance the discussion of the product under review to include further technical, scientific, economic, logistical, and social considerations, in addition to the perspective of stakeholders about the CONITEC’s preliminary recommendation [93]. Such a process also ensures that medications, procedures, and medical devices available through the SUS are both safe and effective and demonstrate an adequate cost–benefit ratio. Along these lines, the HTA has an essential role in the decision-making process for the incorporation of new technologies into the SUS, ensuring an efficient allocation of public resources and that new technology offers tangible clinical benefits.

#### 4.3.2. The Structure of the CONITEC

The CONITEC’s members are responsible for making decisions based on the analysis of reports on the technologies under evaluation and considering patient contributions. From 2011 to 2021, the CONITEC worked with a plenary structure comprising seven representatives from various secretariats of the Ministry of Health, with its executive management entrusted to the Department of Management and Incorporation of Health Technologies. During this period, the CONITEC focused on the development of HTA methodologies to inform decision-making in the Ministry of Health, drawing inspiration from already existing HTA bodies, particularly the U.S. National Institutes of Health (NIH) and the U.K. National Institute for Health and Care Excellence (NICE). However, the CONITEC was not prioritizing the implementation of procedures and processes to ensure effective social participation initially. Such a framework was gradually developed over time, largely due to the efforts of an organized civil society.

The executive secretariat of the CONITEC is still managed by the Department of Management and Incorporation of Health Technologies, and, as established by Decree n. 11,161/2022 [94], the CONITEC is now composed of three thematic committees, with 15 members from institutions broadly representing public health in Brazil, all of whom hold voting rights. The committees are organized into thematic areas: (i) the Products and Procedures Committee, (ii) the Medicines Committee, and (iii) the Clinical Protocols and Therapeutic Guidelines Committee. Despite these structural changes and the creation of thematic committees, Lopes (2024) [95] highlights that representatives of the “public” sector in the CONITEC’s committees are appointed to fixed positions—regardless of the specific topic under analysis—and participate directly in the deliberative process to evaluate each case with voting rights [95]. Furthermore, the individuals designated to represent the “public” are selected by the National Health Council, which represents not only SUS users but also health professionals and the private sector. Accordingly, Lopes suggests that these representatives have diffused interests that may not be directly aligned with each specific incorporation demand and underscores that patients and their families primarily rely on public consultations as their principal avenue for social participation in the process of the incorporation of new technologies into the SUS [95]. Accordingly, concerns persist from patient associations that their contributions are often not adequately considered within the HTA process. This assertion is corroborated by recent studies conducted by Oliveira [81], which highlight the limitations of these current practices.

### 4.4. HTA for Rare Diseases

While the HTA provides established methodologies for evaluating health technologies, there are currently no specific HTA frameworks tailored for the assessment of treatments for rare diseases. Although therapies for rare conditions, such as the CFTR modulators discussed here, demonstrate significant clinical benefits, they present challenges within the HTA framework. Along these lines, the HTA should not be applied without careful consideration of the unique characteristics and complexities involved in the development and evaluation of treatments for (ultra)rare diseases, which magnify the structural weaknesses in the HTA, while exposing the systemic barriers. These challenges stem from factors such as the small patient populations, the lack of long-term outcomes, and the inherent heterogeneity of these diseases. Furthermore, the evidence available to support the clinical and cost-effectiveness of such treatments is often perceived as limited in the context of rare diseases, contributing to a level of uncertainty in decision-making processes [95].

All these challenges have been noticed by family/patient associations and SUS users since the CONITEC faces difficulties in accounting for the unique features of these conditions. This issue has become increasingly salient in the context of contemporary advancements in medical technology, which are marked by innovations that are not only highly personalized and effective but also increasingly expensive. For instance, CFTR modulators offer significant clinical benefits for a specific sub-group of PwCF. However, because CF is a rare disease composed of a relatively small group, these individuals need to mobilize large resources and efforts to ensure access to the most effective treatments available. Such a fact becomes even more challenging in the context of personalized medicine, in which these medications are developed for the most prevalent genetic variants, but the assessment for (ultra)rare variants is impractical via traditional clinical trial designs—the gold standard of evidence-based medicine—due to the small and heterogeneous sample size. This summarizes some of the main tensions involving the incorporation of drugs for rare diseases: (i) excessive prices to the point of representing a challenge to the sustainability of the system’s financing; (ii) evidence of efficacy considered by HTA technologists as weak or moderate, without problematizing that such evidence may be due to the intrinsic features of this population; and (iii) outcomes considered moderate or modest—even though many patient’s testimonies in public consultations indicate otherwise. In this context, judicialization has become a frequent alternative route for families seeking access to these therapies, which can inadvertently fuel litigation and exacerbate disparities in access, thus underscoring the need for more efficient and transparent HTA processes.

Given this scenario, together with constrained investments in healthcare and the growing pressure from various societal sectors to ensure the fiscal sustainability of a unified and publicly funded health system, even disruptive technologies that promise to significantly alter the natural course of a disease encounter numerous obstacles. In the case of CF, the incorporation process of CFTR modulators occurred at different time points, influenced by the shifting political landscape of Brazil. While the organizational structure and submission procedures to the CONITEC are well-established, these frameworks do not fully shield the process from the influence of specific political contexts, as noted by Lopes [95], who describes the CONITEC as “vulnerable to specific political contexts.” This vulnerability introduces significant layers of complexity into the HTA process. Consequently, SUS users—particularly through their representatives, such as patient associations—must dedicate considerable time and resources to understanding and engaging with the process. This involvement is essential to formulate effective strategies for social participation and advocacy from the outset.

From the political context, investments in knowledge production and research are critical to progressing the discourse on social participation, particularly by highlighting issues that have historically been marginalized, overlooked, or ignored within decision-making processes. These issues include the contributions of SUS users, patients, family members, and patient associations during public consultations and HTA processes. Within the domain of health education, fostering discussion forums with patient associations is vital to promote a deeper understanding of these processes and to encourage engagement from all relevant stakeholders, including SUS users, patient groups, family members, healthcare professionals, policymakers, technologists, and industry representatives.

Moreover, in a context where various interests are at play, it is crucial to recognize that the most contentious ethical dilemmas often stem from the exorbitant costs of new medical technologies, which are especially problematic in low- and middle-income countries. Such facts compel policymakers to make difficult decisions about the prioritization of treatments based solely on cost-effectiveness. In this environment, PwCF and their families are confronted with the painful realization that the value of their health and lives is being determined by economic factors. Their suffering is exacerbated not by the disease itself—since treatments are now available—but by their inability to access these treatments due to prohibitive costs. Notably, these treatments are products of years of research, often supported by the financial and logistical contributions of patients and their families, but remain out of reach because of market dynamics. In effect, the accessibility of life-saving treatments is determined solely by market forces, rendering human life subject to the constraints and priorities of the economic system.

## 5. Lay Society Participation in HTA

### 5.1. Patient Organizations as Advocates and Educators

In the health sector, nonprofit organizations often represent individuals with specific diseases, advocate for their rights, and raise societal awareness about health issues. When patients or their families actively contribute to the community, whether in medical–scientific fields, civil society, or public policy, they become valuable allies in advancing the common good [96]. Among the many roles of patient associations, their advocacy in political and social spheres is particularly significant. By representing the interests of patients, they act as a collective voice in public discourse, fostering progress both individually and collectively and upholding the principles of democracy [97]. Moreover, patient associations play a crucial role in health education, particularly in the context of the HTA. They can effectively communicate the complexities of each stage of the HTA process, including the concept and significance of public consultations, and guide how to participate meaningfully. Thus, patient associations fulfill a multifaceted role that ranges from supporting patients and families with treatment and quality of life issues to actively engaging in the development and implementation of public policies.

### 5.2. Patient Experience and Knowledge Production

Rabeharisoa (2017) [98] pointed out that over the past two decades the field of social science research has demonstrated that knowledge production is no longer an exclusive domain of the scientific community’s competencies and prerogatives. We have observed a growing movement, built amidst many tensions, to give visibility to this necessary expansion. Rabeharisoa also indicated that research programs are increasingly incorporating expressions such as “collaborative research”, “stakeholder engagement”, and “interdisciplinary” into their repertoires [98]. This highlights that after a long period of adopting and promoting the slogan “Science and Society”, the European Union has been shifting, to address the democratic deficit of which it is accused, towards emphasizing a perspective that aims to develop a “Science with and for Society”. It is worth noting that all these changes are more easily announced than implemented in the daily practices of the research, investments, and policies involved—for instance, in the provision of new drugs and therapeutic innovations. Nevertheless, despite the opening of institutions towards knowledge constructed by patient associations, they tend to soften and homogenize knowledge from the experience of groups of patients so that it fits within the model established by scientific knowledge. As Rabeharisoa [98] pointed out, this is not about “romanticizing” knowledge from experience, as if it was knowledge closer to reality, because it would be less reductionist than scientific knowledge. We argue that the challenge for patient associations is to understand that knowledge constructed through the experiences of patients and their caregivers also needs to be collected, organized, selected, compared, and analyzed within a broad spectrum of diverse and scattered observations. However, we emphasize that institutions, especially those involved in HTAs, in turn, need to focus more fairly and epistemologically on the knowledge produced from the experiences of patients, family members, and caregivers, in addition to simply expanding the spaces for social participation.

In Brazil, although there has been some progress in this regard, it is still up to managers, mainly representatives at the CONITEC’s committees, to recognize that patients and associations can create tension and organize both the knowledge that is being considered in collective discussions and how this knowledge is being produced [99].

### 5.3. The CF Case: Building Capacity and Advocating for Access

Novas (2006) [100] reported that individuals affected by genetic conditions have become pivotal stakeholders in advancing the health and well-being of others with similar conditions, contributing to the increased generation of biomedical knowledge, the organization of resources for medical research, and the development of new pharmaceutical treatments. However, such a role—particularly in terms of financing research and participating in the design and implementation of clinical trials—is still a distant reality for Brazil. Nevertheless, Brazilian patient associations have been progressively expanding their influence by fostering dialogs with a broad range of stakeholders, including medical societies, public administrators, policymakers, healthcare professionals, academia, legal professionals, researchers, and representatives of the pharmaceutical industry. Through collective efforts, particularly since 2017, the community of PwCF in Brazil, represented by various patient associations, successfully advocated for the incorporation of two out of four CFTR modulators submitted to the CONITEC. This achievement highlights the growing strength of patient advocacy in influencing health policy and decision-making, particularly within the context of rare diseases.

### 5.4. Establishing the Path to CFTR Modulator Access in Brazil

Patient associations have been fundamental in promoting the basic rights of patients, access to quality information about (ultra)rare diseases, and advocating for accurate and early diagnoses and new health technologies. In many countries, these associations expand their scope by actively engaging in research financing and the development of improved drugs. In Brazil, PwCF are assisted and represented by 25 regional patient associations working specifically in their states and two nationwide associations: the Brazilian Association for Assistance to Mucoviscidosis (ABRAM), founded in 1979 [101], and the United for Life Institute (UPV), founded in 2011 [102].

For instance, the UPV was established with the primary goal of strengthening the CF community through initiatives focused on communication, awareness, education, health literacy, and advocacy, thus facilitating the dissemination of fundamental information about the disease for both the scientific and lay society. In the context of the incorporation of CFTR modulators into the SUS, the UPV carried out several initiatives aimed at enhancing the CF community’s understanding of the HTA, advocacy, and public policies, ensuring that everyone could engage meaningfully and effectively. Moreover, the UPV organized forums and symposiums about the HTA and advocacy in the context of CF, bringing together family members, PwCF, caregivers, healthcare professionals, policymakers, researchers, and industry representatives to discuss key issues affecting the CF community.

Through training, education, and coordination, the UPV, in collaboration with other CF associations and stakeholders, has played a pivotal role in addressing the challenges faced by the CF community during the evaluation processes of the CFTR modulators submitted to the CONITEC. This coordinated effort contributed to structuring advocacy strategies and stakeholder engagement, enabling the more effective navigation of the procedural and institutional complexities inherent to the HTA process. Alongside other national and international CF organizations, the UPV led targeted public campaigns and actions demanding a significant reduction in the high prices set by the pharmaceutical industry for these new health technologies. Indeed, these actions were crucial in making the incorporation of these treatments into the SUS more financially sustainable. Table 3 depicts several actions taken to qualify social participation in HTA processes and the generation of real-world data for articulation and advocacy actions.

These actions, together with the quality of the clinical evidence of ETI, ensured a favorable decision for the incorporation of this therapy into the SUS for PwCF harboring F508del on at least one allele. However, it should be stressed that a key decisive factor for such an outcome was the substantial price reduction after the public consultation. This leads us to reflect on how the composition of health technology prices—especially high-cost medications—needs to be addressed and discussed with much more transparency with the whole of society. In this field specifically, we have a long way to go, as researchers have paid little attention to the composition of drug prices. As suggested by Rabeharisoa [98], this issue remains an almost inaccessible research topic. However, we know that working in the field of rare diseases has become an especially lucrative business. Patient associations have increasingly taken a stand and reported the exorbitant prices charged for these drugs and the devastating effects on the lives of thousands of patients and their families due to the delay or even impossibility of accessing these new drugs.

Along these lines, the UPV and other CF associations in Brazil have spearheaded numerous social advocacy campaigns. With the support of CF assistance organizations across the nation, the UPV launched the #RegistraVertex campaign in December 2019 (i.e., just over a month after the FDA approved ETI) to urge the pharmaceutical industry to apply for the drug’s registration in Brazil. Subsequently, the #TrikaftaJá campaign was initiated to monitor the regulatory approval process, along with actions demanding a reduction in the drug’s price and its inclusion into the SUS’ reimbursement list [35].

The call for the affordable pricing of treatments that is aligned with the economic realities of each country is a global concern, reinforced by international movements like Vertex Save Us, which advocates “*for there to be a new era in Cystic Fibrosis, there must be equitable access to CFTR modulators”* [103]. In parallel, the ABRAM led an initiative for the compulsory licensing of ETI in Brazil, submitting a letter to the Brazilian Minister of Health requesting that CFTR modulators be declared of public interest, thereby enabling broader access to the treatment through compulsory patent licenses [104]. These and other actions carried out in Brazil corroborate the global demand of the CF community, which considers that CFTR modulators are the best treatment available, but the high prices of these drugs make them almost inaccessible outside the high-income countries [26]. In addition, there is evidence in the literature indicating that the costs to produce CFTR modulators can be 85% lower than their current prices [26]. Therefore, price negotiations are urgently needed so that CFTR modulators can be accessible to all eligible PwCF worldwide.

As a result of efforts to foster qualified lay society participation in the CONITEC’s public consultations, a case study conducted in 2023 revealed that the public consultation on the IVA monotherapy (held in 2020) received 10,417 contributions through the experience and opinion form, including 370 from PwCF [81,85]. According to the CONITEC’s official monitoring dashboard [105], this was the fifth-highest number of contributions received across all consultations. In the case of ETI, following the implementation of a new submission form, 3140 contributions were received, of which 2935 were experience-based testimonials. While these numbers reflect a significant degree of civil mobilization and engagement, it should be stated that the HTA process prioritizes the quality of contributions, particularly those grounded in real-world evidence from individuals already using the technology under evaluation. Along these lines, patient participation can serve as a crucial corrective to many limitations in the HTA process, including counteracting utilitarian biases, bridging sociotechnical gaps, and enhancing legitimacy and trust. Nevertheless, even when high-quality data are presented, patient participation does not automatically translate into an effective influence on decision-making, as highlighted by this study and the broader literature on the limitations of fully integrating societal input into HTA processes.

Finally, while CF patient associations in Brazil have played a key role in education, advocacy, and social participation in HTAs, it is important to acknowledge ethical and structural challenges. Budgetary constraints, a reliance on volunteers, regional disparities, and the proximity to the pharmaceutical industry raise concerns about sustainability, autonomy, and representativeness. These aspects reinforce the need for transparency and inclusive strategies aligned with critical perspectives from health sociology and bioethics.

## 6. Ethics in HTA: A Subject That Needs Further Attention

It is widely acknowledged that technological innovations, particularly in the fields of biomedicine and biotechnology, are reshaping our perceptions of what is considered “normal” and “natural.” In this context, biotechnology has the potential to disrupt not only our conceptions of health and well-being but also our understanding of what it means to have a good life. However, biotechnology raises concerns that extend beyond traditional debates about safety and the equity of access, delving into questions regarding the types of human beings and societies we are creating through our decisions in this field.

In many discussions, technology is often portrayed as something separate from society, as if it exists in a pre-social space and is therefore not influenced by political discourse or power dynamics. However, scholars in Science and Technology Studies, such as Petersen [106], argue that technologies are inherently social. Technologies are produced, gain meaning, and reflect societal values and interests through social interactions. With this perspective in mind, the ethical issues surrounding the decision-making about the incorporation of new technologies, particularly in the health sector, present a complex, multifaceted, and highly significant discussion—one that is often contentious. There is a substantial body of literature that addresses the integration of ethical concerns into HTAs [107,108,109,110]. These works collectively underscore that healthcare is a moral enterprise, with ethics playing a central role in framing these discussions. Given the increasing complexity of health-related decision-making, the fundamental questions remain: What is the right course of action? What is the best decision?

These questions become even more challenging in the context of rare diseases such as CF, and the answer is highly contingent on the ethical framework applied. As Picavet and collaborators highlight, one ethical perspective is grounded in principles of equity, individual rights, and non-abandonment, which prioritize preserving the right of everyone to a dignified life and access to the best available treatment [111]. In contrast, a utilitarian perspective, which seeks to maximize societal health outcomes, may prioritize the collective good, often at the expense of a smaller, more marginalized group. Accordingly, while the HTA has been essential for evidence-based policymaking, its utilitarian foundation (e.g., neglect of equity and justice, undervaluing individual needs and moral distress), lack of sociotechnical context (e.g., resource constraints and inequity), and methodological limitations (e.g., focus on short-term effects, exclusion of patient preferences, and static assessments) can lead to ethically contentious and socially disconnected decisions [112].

We are currently experiencing a disruptive and transformative period in CF treatments, largely due to CFTR modulators. These medications have significantly improved outcomes and reduced suffering for genotype-eligible PwCF. However, this progress has also exposed and intensified pre-existing disparities in CF care globally. Access to an early diagnosis and the best available treatment for CF has never been merely a matter of scientific or medical advancement. It is, at its core, a matter of geopolitics, race, ethnicity, economic development, and the social determinants of health [113]. These issues highlight the immense inequity faced by PwCF in low- and middle-income countries, especially in regions outside of the U.S. and Central Europe. Addressing these challenges requires a collaboration between governments, patient organizations, legal professionals, economists, and the pharmaceutical industry responsible for drug patents to find solutions that are both equitable and sustainable. Such solutions should prioritize patient access to life-saving treatments without destabilizing the health budgets of low- and middle-income countries. This includes confronting complex issues related to international economic agreements, intellectual property rights, technology transfer, compulsory licensing, and the pricing of drugs relative to their production costs.

Despite the availability of multiple ethical perspectives, the utilitarian viewpoint often prevails in decision-making processes within HTAs, especially in the context of rare diseases. This perspective argues that the allocation of limited resources to benefit a small group of patients may not be in the best interest of society as a whole. Furthermore, the utilitarian framework may deem funding research into rare diseases unethical. As Hofman and co-authors note [108], utilitarianism is central to effectiveness, safety, and cost-effectiveness analyses commonly performed in HTAs. While utilitarianism is an influential ethical perspective, it can have devastating consequences for minority or marginalized groups (e.g., patients with (ultra)rare diseases living in underprivileged regions), whose needs may be marginalized in favor of maximizing benefits for the majority. Moreover, the monopolistic nature of the market, with exorbitantly priced life-saving drugs sold by a single manufacturer, raises serious ethical concerns regarding sustainability and fairness. This situation is ethically untenable, as it may prevent many patients from accessing essential treatments due to prohibitively high costs.

Brazil has emerged as a leader in Latin America in providing access to the ETI (marketed as Trikafta^®^) for CF through its public healthcare system. However, the discussions surrounding this incorporation were fraught with challenges, particularly regarding the economic impact on the healthcare system. The final decision in favor of the treatment was influenced by several key factors: an engaged and mobilized community, including patient associations participating in public consultations and campaigns for drug price reductions; social advocacy for the compulsory licensing of CFTR modulators; the involvement of government officials, even before the evaluation process began, signaling a willingness to engage in dialog; and the active participation of medical professionals through organizations such as the Brazilian Cystic Fibrosis Study Group (GBEFC) and the Brazilian Society of Pulmonology. These factors created a favorable environment for the approval of the medication. However, it is important to note that without a significant reduction in the price set by the manufacturer, as highlighted before in this article, this broad-based advocacy would not have been sufficient to secure approval and subsequent dispensing. This situation raises concerns for the future, as approximately half of the CF population in Brazil remains ineligible for the available CFTR modulators, and more advanced technologies (e.g., genetic therapies) will be even more expensive. Addressing the ethical, financial, and systemic challenges in the context of rare diseases like CF requires an ongoing commitment and collaboration from all stakeholders involved.

## 7. Outlook and Concluding Remarks

Tremendous efforts have been made in Brazil to foster a more horizontal dialog among the CF community, healthcare professionals, medical teams, and researchers involved in both patient care and fundamental research. Central to this initiative has been the establishment of collaborative spaces at conferences and symposia, where patient representatives, researchers, and healthcare professionals can collectively develop agendas and engage in meaningful dialog on common interests. Additionally, the collaboration between the GBEFC and patient associations has been instrumental in fostering a deeper understanding of the diverse roles that doctors, patients, families, and representatives must play in the process of incorporating new technologies into the SUS.

Another significant development in this process was the creation of formal spaces for dialog by the Ministry of Health, through its Secretariat of Science, Technology, Innovation, and the Economic–Industrial Complex of Health. These spaces facilitated the sharing of critical data, such as that from the Brazilian Cystic Fibrosis Registry, which informed the HTA process and highlighted issues that are often overlooked in traditional technical analyses.

Equally important was the reduction in treatment costs, achieved through negotiations with the pharmaceutical industry and, crucially, through discussions on the potential for compulsory registration, which prompted an examination of pricing transparency and its implications. These efforts were made possible through a series of meetings, symposia, and forums in which stakeholders from various sectors, each with distinct areas of interest, contributed their expertise and perspectives on complex issues and challenging solutions. One possible solution to the cost-effectiveness thresholds would be to adopt a rare-disease-specific cost-effectiveness criterion, based on the medications’ price list worldwide. Or even, the pharmaceutical industry could contribute with a financial risk, or risk-sharing with the public health system, for the benefit of the patients. This article not only chronicles the journey of incorporating CFTR modulators in Brazil but also offers a critical reflection on this process. The insights provided are intended to serve as a resource for other communities facing similar challenges in ensuring sustainable, timely, and equitable access to the best possible treatments for those in need.

## Figures and Tables

**Table 1:** Approval date of CFTR modulator drugs by different national regulatory agencies.

Country Region	Regulatory Agency	IVA	LUM/IVA	TEZ/IVA	ELX/TEZ/IVA	VNZ/TEZ/dIVA
U.S.	Food and Drug Administration (FDA)	Jan/2012	Jul/2015	Feb/2018	Oct/2019	Dec/2024
European Union	European Medicines Agency (EMA)	Jul/2012	Nov/2015	Oct/2018	Aug/2020	Jul/2025
U.K.	Medicines and Healthcare Products Regulatory Agency (MHRA)	Jul/2012	Nov/2015	Oct/2018	Aug/2020	March/2025
Canada	Health Canada	Nov/2012	Jan/2016	Jun/2018	Jun/2021	Jul/2025
Australia	Therapeutic Goods Administration (TGA)	Jun/2019	Aug/2018	Mar/2018	Mar/2021	Submitted
Brazil	Brazilian Health Regulatory Agency (ANVISA)	Sept/2018	Jul/2018	Jan/2020	Mar/2022	Not Registered
Argentina	National Administration of Drugs, Food, and Medical Devices (ANMAT)	Not registered	Not registered	Not registered	Not registered	Not Registered
South Africa	South African Health Products Regulatory Authority (SAHPRA)	Not registered	Not registered	Not registered	Not registered	Not Registered

Abbreviations: ELX—elexacaftor; dIVA—deutivacaftor; IVA—ivacaftor; LUM—lumacaftor; TEZ—tezacaftor; and VNZ—vanzacaftor.

**Table 2:** The CFTR modulators, age of initiation, and variants approved by the ANVISA in Brazil.

CFTR Modulator (Tradename)	Age (Years)	Incorporated into SUS	Variants
IVA (Kalydeco^®^)	≥6	Dec/2020	At least one copy of the gating mutations—G551D, G1244E, G1349D, G178R, G551S, S1251N, S1255P, S549N, or S549R—and patients aged 18 years or older with one copy of the R117H mutation
LUM/IVA (Orkambi^®^)	≥6	Not incorporated	Two copies of F508del
TEZ/IVA (Symdeko^®^)	≥12	Not incorporated	Two copies of F508del or one copy of F508del and a residual functional variant on the second allele (P67L, D110H, R117C, L206W, R352Q, A455E, D579G, 711+3A>G, S945L, S977F, R1070W, D1152H, 2657+5G>A, 3140-26A>G, and 3717+12191C>T)
ELX/TEZ/IVA (Trikafta^®^)	≥6	Sep/2023	At least one copy of F508del

Abbreviations: ELX—elexacaftor; IVA—ivacaftor; LUM—lumacaftor; and TEZ—tezacaftor.

**Table 3:** A summary of the social engagement journey (2012–2024): the path to incorporating CFTR modulators into the SUS.

Goal	Activities Led by the UPV
Community Education and Engagement	>300 educational posts on the website. >50 articles “about HTA and the incorporation process”. >45 informative podcasts. >100 educational live sessions. Special live video with the CONITEC to explain HTA process.
Preparing CF Patient Organizations in Brazil	6 national development meetings with workshops on HTA, advocacy, and social participation. Approximately 20 CF associations and 34 participants per edition.
Research and Data Generation	3 special research booklets on the process of technology incorporation into the SUS. “Qualifica SUS” research with patient associations across Brazil. Technical contribution in public consultation.
National Events about the HTA Process	Conduction of three editions of the Brazilian HTA Forum for Rare Diseases. The first edition was held in 2022 and the second and third in 2023 and 2025, respectively, in a hybrid format, with live streaming and simultaneous translation. A horizontal event featuring participation from the Ministry of Health, CONITEC, the Legislative Branch, patient associations, the pharmaceutical industry, and the patients.
Strategic Meetings to Engage the CF Community: Advocacy and Stakeholder Engagement Preparatory Actions for Public Consultation	Dozens of meetings and dialogs with the Ministry of Health, patient organizations, medical societies, legislative representatives, and the industry, especially advocating price reductions. Dedicated website for each public consultation, featuring videos, step-by-step guides, and additional resources.

## Data Availability

Data sharing is not applicable. No new data were created or analyzed in this study.

## Abbreviations

The following abbreviations are used in this manuscript:

ABRAM	Association for Assistance to Mucoviscidosis
ANMAT	Argentinian National Administration of Drugs, Food and Medical Devices
ANVISA	Brazilian Health Regulatory Agency
CF	Cystic Fibrosis
CFTR	Cystic Fibrosis Transmembrane Conductance Regulator
CMED	Chamber for the Regulation of the Drug Market
CONITEC	Brazilian National Commission for the Incorporation of Technologies into SUS
dIVA	Deutivacaftor
ELX	Elexacaftor
EMA	European Medicines Agency
ETI	Elexacaftor/Tezacaftor/Ivacaftor
FDA	U.S. Food and Drug Administration
GBEFC	Brazilian Cystic Fibrosis Study Group
HTA	Health Technology Assessment
IVA	Ivacaftor
MHRA	Medicines and Healthcare Products Regulatory Agency
PwCF	People with Cystic Fibrosis
SAHPRA	South African Health Products Regulatory Authority
SUS	Brazilian Unified Health Care System
TEZ	Tezacaftor
TGA	Australian Therapeutic Goods Administration
UPV	United for Life Institute
VNZ	Vanzacaftor
